# Constipation in autistic people and people with learning disabilities

**DOI:** 10.3399/bjgp22X720077

**Published:** 2022-07-01

**Authors:** Christina Maslen, Rebecca Hodge, Kim Tie, Richard Laugharne, Kirsten Lamb, Rohit Shankar

**Affiliations:** NHS South, Central and West, Eastleigh.; Clinical Services Programme Lead: Mental Health, NHS South, Central and West, Eastleigh.; NHS South, Central and West, Eastleigh.; Cornwall Partnership NHS Foundation Trust, Truro; Cornwall Intellectual Disability Equitable Research (CIDER), University of Plymouth, Peninsula School of Medicine, Truro.; Honorary Associate Professor CIDER, Learning Disability Special Interest group, Royal College of General Practitioners, London.; Cornwall Partnership NHS Foundation Trust, Truro; Cornwall Intellectual Disability Equitable Research (CIDER), University of Plymouth, Peninsula School of Medicine, Truro.

## INTRODUCTION

This article gives an overview of constipation and related concerns in people with learning disabilities (LDs) and/or autistic people. It provides recommendations to primary care to help address this issue. It furthers the evidence and importance of recognising and managing constipation as outlined in national guidance.^1^

Public Health England and the Office for National Statistics estimate that approximately 2.16% of adults and 2.50% of children in the UK have an LD. Autistic spectrum condition (ASC) is reported in approximately 2% of adults and children. It is considered that 30%–70% of people who have an ASC will also have an LD.^2^

People with LDs die on average 20 years earlier than the general population and suffer much higher rates of physical morbidities.^3^ The Learning Disabilities Mortality Review (LeDeR) found that 23% of deaths identified constipation as a long-term health problem and 33% were on prescribed laxatives.^1^ Another study highlighted that, while only 0.5% of the general population are on regular laxatives, this figure is 25.0% of the LD population.^4^

There are numerous causes for this tendency to constipation. There may be neurological and genetic causes of disordered bowel function. People with LDs may struggle to communicate symptoms and discomfort. Reduced mobility can be a problem especially if the LD is severe. Fluid and diet intake may be not optimum, especially for those with dysphagia. Medication side effects can include constipation, particularly medication with anticholinergic side effects, including opioids and psychotropic and anti-seizure medication.

Autistic people (like people with LDs) experience more health inequalities compared with the general population, including higher rates of sleep problems, epilepsy, sensory impairments, allergies, autoimmune disorders, and obesity.^5^ Autistic people have a two to three decades’ decreased life expectancy and heightened all-cause and injury mortality.^6^ Atypical eating behaviours are common in those with ASC, with the most frequent being limited food preferences, food texture hypersensitivity, pica, and other unusual patterns, such as eating only one brand of food. Atypical eating behaviours are more common in ASC than in other developmental disabilities and occurrence may be as high as 70.5%.^7^

## PREVALENCE OF CONSTIPATION AND ASSOCIATED DISORDERS IN LD OR ASC POPULATIONS

### LDs and constipation

A systematic review of 31 studies published between 1990 and 2016 found that people with an LD were more likely to suffer from constipation than people without an LD.^8^ The review found that, across studies, prevalence was high, with 21 studies reporting rates of over 33% and 14 studies reporting rates of 50% or more. Over 12 months, laxative prescriptions were received by 25.7% of people with an LD compared with 0.1% of people without an LD. Constipation was registered as a health problem for nearly 60% of people with profound learning and multiple disabilities, 65% of whom had been prescribed laxatives in the previous year. Age was not consistently associated with constipation, but physical inactivity was. Studies published after this review include a regional Scottish study, which found that constipation, gastro-oesophageal reflux disease, and dysphagia were the three gastrointestinal (GI) disorders that were in the top 20 physical health conditions for people with LDs, with prevalence of 33.8%, 14.5%, and 12.9%, respectively.^9^ Constipation was the fourth most prevalent physical health condition for the whole cohort overall, occurring at a rate of 33.8%. Among those with Down’s syndrome the rate was 24.1% compared with 36% for those in the cohort with an LD but without Down’s syndrome. A Spanish study exploring health status in a large representative and stratified sample of people with LDs found that constipation was highly prevalent among the participants, with rates ranging from 25% in the 18–25-year-old age group to 38% in the over-65s.^10^ This study also found that prevalence is associated with LD severity level, with lower rates in those with mild-to-moderate LDs, rising to 56% in those with severe-to-profound LDs. A systematic review of Down’s syndrome and bladder and bowel dysfunction found that 90% had functional constipation.^11^

### ASC and constipation

A US study suggested that children with ASC may be at a higher lifetime risk of constipation and of needing medication as compared with children with other development behavioural disorders such as an LD.^12^ Constipation/chronic constipation/functional constipation was most frequently cited, appearing in 12 of the studies reporting GI abnormalities for autistic people. This review confirmed the results of an earlier meta-analysis, which showed that GI disorders in children with ASC were more prevalent than in healthy children, with higher rates of constipation (odds ratio 3.86, 95% confidence interval [CI] = 2.23 to 6.71).^13^ Another study found a high rate (median 46.8%) of any GI symptoms in those with ASC across 144 studies. The most common of these appeared to be chronic constipation, with a median prevalence across studies of 22% (range 4.3%–45.5%).^14^ The GI problems most reported in ASC are chronic constipation, abdominal pain with or without diarrhoea, and encopresis because of constipation.^15^ A narrative systematic review of 18 studies published between 2005 and 2017 found that 15 studies reported increased prevalence of GI symptoms in ASC patients, with constipation and diarrhoea accounting for the highest rates.^16^

### Associated problems with constipation, particularly in ASC

Estimates for the prevalence of any GI problems with ASC vary between 9% and 70% but may even be as high as 91%.^17^ The large differences in prevalence rates between individual studies may be due to the need to rely on parental report, inconsistent constipation definitions, suboptimal or lack of control groups, and heterogeneous study populations, as well as population stratification variability and variable criteria in defining GI disorders. In a small prospective case–control study between participants with ASC and those without, significantly higher rates of faecal incontinence (36.3%) and constipation (68.1%) were identified in those with ASC.^18^ A significant association with mood disorders was also found.^18^

## HOSPITALISATION OF INDIVIDUALS WITH AN LD AND/OR ASC FOR GASTROINTESTINAL PROBLEMS INCLUDING CONSTIPATION

An English database study identified key causes of hospitalisation for people with LDs.^19^ The study found that the most common were epilepsy, influenza pneumonia, aspiration pneumonitis, dehydration, gastroenteritis, and constipation. A US database emergency department review on constipation identified that children with ASC were more likely than those with no or other long-term conditions to visit an emergency department (ED) (1.9% versus 0.6% versus 0.9%; *P*<0.001).^18^ They were also more likely to be admitted to the hospital (15.0% versus 10.6% versus 1.2%; *P*<0.001) after the ED visit.^20^ The National Emergency Laparotomy Audit 2020 indicated 307 patients as having either an LD and/or ASC out of the 24 823 patients who had emergency laparotomy surgery for GI problems.^21^ Of all patients with an LD and/or ASC, 68.0% were admitted to critical care, compared with 62.9% overall. Length of stay was longer for patients with an LD and/or ASC compared with overall length of stay (mean duration: 20 days versus 15 days). The 30-day mortality in this group was 11.7%. There is limited evidence of over-representation of constipation-induced volvulus and pseudo-obstruction in the LD population. Unfortunately, the full details for those with an LD and/or ASC who had a laparotomy for pseudo-obstruction/acute volvulus caused by constipation were not available to better understand this. Another major medical complication of constipation is faecal impaction. A study explored the prevalence of faecal impaction as a complication of constipation in residents in 34 general (non-LD) nursing homes.^22^ The prevalence of chronic constipation was 70.7% (95% CI = 67.3 to 74.1), and faecal impaction was found in 47.3% of those. The study suggested that both uncontrolled and controlled constipation, total number of medications, and reduced functional capacity were independent risk factors for faecal impaction. No similar studies are available in people with LDs.

## MORTALITY IN LD AND/OR ASC POPULATIONS DUE TO CONSTIPATION

A UK population-based study, while not reporting data solely for constipation (data were reported by International Classification of Diseases, 10th revision [ICD-10] chapter groupings of conditions), noted that both constipation and bowel incontinence were predictors of death.^23^

However, reports of death due to constipation in people with LDs and/or autistic people rarely feature in the academic literature. It has been suggested that the way deaths are recorded and the hospital episodes are coded could contribute to obscuring the detail in identifying complex and multifactorial underlying cause(s) of both admission and death. Studies investigating admissions or mortality in people with LDs and/or autistic people tend to use ‘umbrella’ ICD-10 chapter groupings of conditions rather than drilling down to individual condition. A systematic review on people with LDs examining the accuracy of the medical certificates of cause of death (MCCD) found significant concerns about the accuracy on MCCD reporting.^24^ A major issue was the under-reporting of an LD and/or ASC on the MCCD. Even when reported, an LD and/or ASC were commonly put as the main underlying cause of death. This causes concern on two grounds. One, there needs to be recognition on MCCD of the contributory effect of a person’s LD and/or ASC. Two, an LD and/or ASC is never the cause of death. The actual cause of death such as constipation or seizures needs to be captured.

Anecdotal reports of constipation being a contributory factor to death are featured in the LeDeR.^3^ Other evidence is from impactful case reports and non-UK literature. An inquest into the death from constipation of a 33-year-old man with Down’s syndrome complicated by Hirschsprung syndrome was widely reported by the UK press in 2018.^25^ A sanitary towel was found in the rectum of another case, thought to be a desperate attempt to stop the prolonged, severe diarrhoea she was experiencing. In another, a young man showing unusual behaviour for several days, possibly experiencing discomfort and pain, and unable to communicate this distress to others, died from constipation complications.^26^ A further two cases describe sudden death in two women, one with ASC and one with an LD.^27^ Both suffered from chronic constipation. Subsequent post-mortem computerised tomography (CT) scans and the autopsies revealed giant faecalomas, which led to death. Their reduced intellectual abilities and the impact this had on communication were considered key elements of delayed medical treatment and ultimately their death. In all cases, death could have probably been prevented with timely medical attention and intervention.

## DISCUSSION

This overview has found that there is significant and consistent evidence, nationally and internationally, that the prevalence of constipation in particular and related GI issues in general in people with LDs and/or autistic people is significantly higher than among the general population. Estimated prevalence rates do vary depending on the sample and methods used in the studies. Differences in defining the population and constipation itself between studies make the estimates of prevalence rates imprecise. The evidence suggests that people with LDs and/or autistic people have more complex needs generally leading to more frequent admission to hospital than the general population. There is evidence that constipation, along with other GI problems, is one of the key causes of avoidable emergency admissions to hospital in this population, although precise figures are not known. Reports of deaths due to constipation, or rather its sequelae such as constipation-induced volvulus and pseudo-obstruction, are few. It may be that data solely taken from the MCCD are inadequate for understanding the mortality experience in this population especially given it is under-reported and the manner in which it is presently captured.

### Implications for primary care

Constipation and GI problems are common in people with LDs and/or autistic people, and may have unusual presentations. Constipation may present with behaviour change, stopping eating, and changed sleep pattern, for example. Active prevention and management are justified because the evidence shows they cause high morbidity, mortality, and hospitalisation rates. The delayed and/or unusual presentation is one of the critical problems that cause acute hospitalisation. Primary care-led regular reviews alongside constipation-led health promotion and prevention strategies, including engagement and education of people with LDs, their families, and carers, should be a priority.

A focused review of bowel health should be an essential part of Annual Health Checks. Those with chronic problems require in-depth assessment, including medication review to exclude iatrogenic causes. Detailed evidence of bowel habits should be sought in advance from the person and/or their carers.

Constipation caused by the patient’s biological disposition needs active management to prevent harm. Box 1 provides an outline of what good practice in primary care could look like. Proactive implementation of such good practice along with suitable education of patients and their carers could go a long way to reduce diagnostic overshadowing, future harm, and death.

**Box 1.** What to consider as a GP when seeing a person with an LD and/or an autistic person with chronic constipation or other GIT concerns

**What to do**	**Who is best placed to help**
Ensure bowel issues are actively reviewed during the annual health check	GP/practice nurse/preparation by person and carers/local LD service may have pre-health check preparation guides
Review any long-term use of laxatives with patients/carers to identify possible deprescribing; consider interventions that offer holistic medicines understanding and support around appropriate use	GP/practice pharmacist
Regularly review long-term/repeat medications, especially those that increase the anticholinergic burden	GP/practice pharmacist
Prescribing of psychotropic medication is common in this population — many have anticholinergic side effects. Seek specialist support on reviewing psychiatric prescribing and its bowel impact	Specialist LD service or psychiatrist working with people with LDs and/or ASC
Consider providing a proactive lifestyle plan to include healthy bowel management plan; diet review; activity review; and fluid intake review	GP/community nurses/dietitian/primary care liaison nurses/LD specialist team
Bowel problems may present with behaviour or personality change, be triggered by change in routine, and be linked with other morbidities — consciously avoid diagnostic overshadowing and arrange specialist referral for detailed evaluation when needed	Hospital liaison services, GIT specialists, and LD specialist services to enable appropriate, timely investigations and interventions
Education and training of the patient, their family, and carers should be accessible and person centred. Communicate any relevant information in a manner the individual can understand	The LD specialist service could support identification and, where needed, development of such resources and tailoring them to the individual

*ASC = autism spectrum conditions. GIT = gastrointestinal tract. LD = learning disability.*

### Implications for research and policy

Reliable monitoring systems to identify deaths caused by constipation is an urgent requirement. This will allow for better understanding and research of the different and wide-ranging factors influencing multimorbidity along with the potential for reducing iatrogenic harm. This includes service delivery, training, and education to the patient’s genetic vulnerability. While there are national-level information and resources such as ‘Poo matters’, it is unclear what the impact of these initiatives have been.^28^ It might be useful to have a common source for good practice and national guidance leaflets. Consideration could be given then to using implementation science as a strategy to promote and examine the uptake of these resources.
